# The effect of differences in viewing environment and method on physiological responses and perceptual stability while viewing three-dimensional images

**DOI:** 10.1265/ehpm.25-00418

**Published:** 2026-08-29

**Authors:** Yasuyuki Matsuura, Shiyu Hitomi, Sota Hibino, Hiroki Takada

**Affiliations:** 1 Department of International Studies and Communications, Faculty of Glocal Policy Management and Communications, Yamanashi Prefectural University, 5-11-1 Iida, Kofu, Yamanashi 400-0035, Japan; 2 Department of Fundamental Engineering for Knowledge-Based Society, Graduate School of Engineering, University of Fukui, 3-9-1 Bunkyo, Fukui-city, Fukui 910-8507, Japan; 3 Department of Mechanical and Systems Engineering, Faculty of Engineering, University of Fukui, 3-9-1 Bunkyo, Fukui-city, Fukui 910-8507, Japan

**Keywords:** Stereoscopic imaging, Illuminance, Color temperature, Hysteresis, Light adaptation, Visual perception, Pursuit vision, Peripheral vision, Physiological responses

## Abstract

**Background:**

Stereoscopic (3D) viewing can induce motion sickness and visual discomfort under specific environmental conditions. Light adaptation is not always linear; preceding light exposure influences subsequent physiological responses (hysteresis). However, hysteresis in 3D viewing has rarely been studied.

**Objective:**

This exploratory study examined whether physiological responses exhibit hysteresis—history-dependent asymmetries between increasing and decreasing illuminance phases—under combinations of low illuminance, color temperature, and viewing mode.

**Methods:**

Eight healthy adults (age 21.75 ± 0.97 yr) were exposed to stereoscopic video under eight conditions (2 color temperatures × 2 illuminance-change directions × 2 viewing modes). Illuminance was varied in stepwise fashion (10–260 lx). Electroencephalogram (EEG; Fp1/Fp2), electrocardiogram (ECG), and Stabilometry were recorded. Repeated-measures ANOVAs with Benjamini–Hochberg FDR correction and paired Hedges’ g effect sizes were computed.

**Results:**

Large hysteresis effects were observed for postural sway (Hedges’ g = 0.66–1.01) and moderate effects for theta-band EEG (|g| ≈ 0.5–0.65), particularly in peripheral vision under low illuminance. Stabilometry was significantly greater at 10 lx compared to higher illuminances (p < 0.05). The interaction between illuminance and illuminance-change direction was significant for total locus length per unit area (L/A) in peripheral vision under daylight color.

**Conclusions:**

Postural control, but not autonomic function, exhibited strong history dependence in response to illuminance changes during 3D viewing. Findings suggest minimum illuminance guidelines (∼80–100 lx) and consideration of illuminance-ramp rates for VR safety and 3D content design.

## 1. Introduction

In recent years, three-dimensional (3D) imaging has become increasingly pervasive across entertainment, medical technology, education, and virtual or augmented reality (VR/AR) applications. However, under certain viewing conditions, viewers frequently report adverse symptoms such as visually induced motion sickness, eye strain, headaches, dizziness, and nausea—collectively recognized as a significant public health concern [1, 2]. Although motion sickness tends to occur more frequently during 3D viewing than during conventional 2D viewing, its underlying mechanisms and effective countermeasures remain insufficiently understood.

Human visual perception relies on distinct visual pathways: central vision for detailed recognition, peripheral vision for wide-field motion detection, and smooth pursuit viewing for tracking moving objects [3–5]. Peripheral vision, supported primarily by rod-rich retinal regions sensitive to low illuminance, plays a crucial role in dynamic 3D perception but has been associated with higher motion sickness risk compared with smooth pursuit viewing [6].

In addition to viewing mode, the lighting environment—encompassing both illuminance and color temperature—substantially influences visual comfort, physiological load, and autonomic function [7–9]. Light adaptation is not always instantaneous or linear: preceding light exposure influences subsequent sensitivity, a phenomenon historically described as light-dark adaptation or hysteresis [10, 11].

Recent literature suggests that when illuminance or color temperature changes, perceptual state transitions may occur abruptly rather than gradually at thresholds, consistent with catastrophe theory [12]. Such history-dependent responses imply that physiological load and discomfort depend not only on current levels but also on approach direction (higher/lower).

However, empirical evidence for hysteresis in 3D viewing remains sparse [13, 14]. To our knowledge, no prior work has systematically investigated asymmetric (hysteretic) patterns under combined low illuminance (10–260 lx ascending/descending), color temperature (incandescent vs. daylight), and viewing mode (peripheral vs. pursuit).

This exploratory study tested whether physiological indicators (Electroencephalogram (EEG), Electrocardiogram (ECG), Stabilometry) during stereoscopic 3D viewing exhibit hysteresis, specifically differing responses between increasing (10→260 lx) and decreasing (260→10 lx) illuminance trajectories. We measured multi-modal signals from eight healthy male adults across eight conditions (2 × 2 × 2), guided by photoreceptor adaptation asymmetry and catastrophe theory [15, 16].

## 2. Methods

### 2.1 Experimental methods

In this experiment, eight young men with normal binocular stereopsis (mean ± standard deviation (SD): 21.75 ± 0.97 years) participated in viewing stereoscopic images. This study was conducted with the approval of the Research Ethics Committee of the Department of Intelligent Systems Engineering, Graduate School of Engineering, University of Fukui (H2022001). Participants were fully informed of the purpose of the experiment in advance, and data collection was conducted after obtaining their consent. EEG measurements were taken using a band-type telemetry-based 2-electrode EEG device (Neuro Sky), ECG measurements were taken using Cardio-stick (Neuro Sky), and Stabilometry measurements were taken using a Balance Wii Board (Nintendo). The experimental data used in this study are identical to those reported in the undergraduate theses of co-authors Hitomi and Hibino [17, 18].

The experiment was conducted under eight conditions: two lighting conditions (incandescent color [2800K] and daylight color [5100K] LED lighting), two illuminance change conditions (increase and decrease processes), and two viewing methods (pursuit and peripheral). These conditions were conducted in a random order to account for the order effect.

**Fig. 1:**
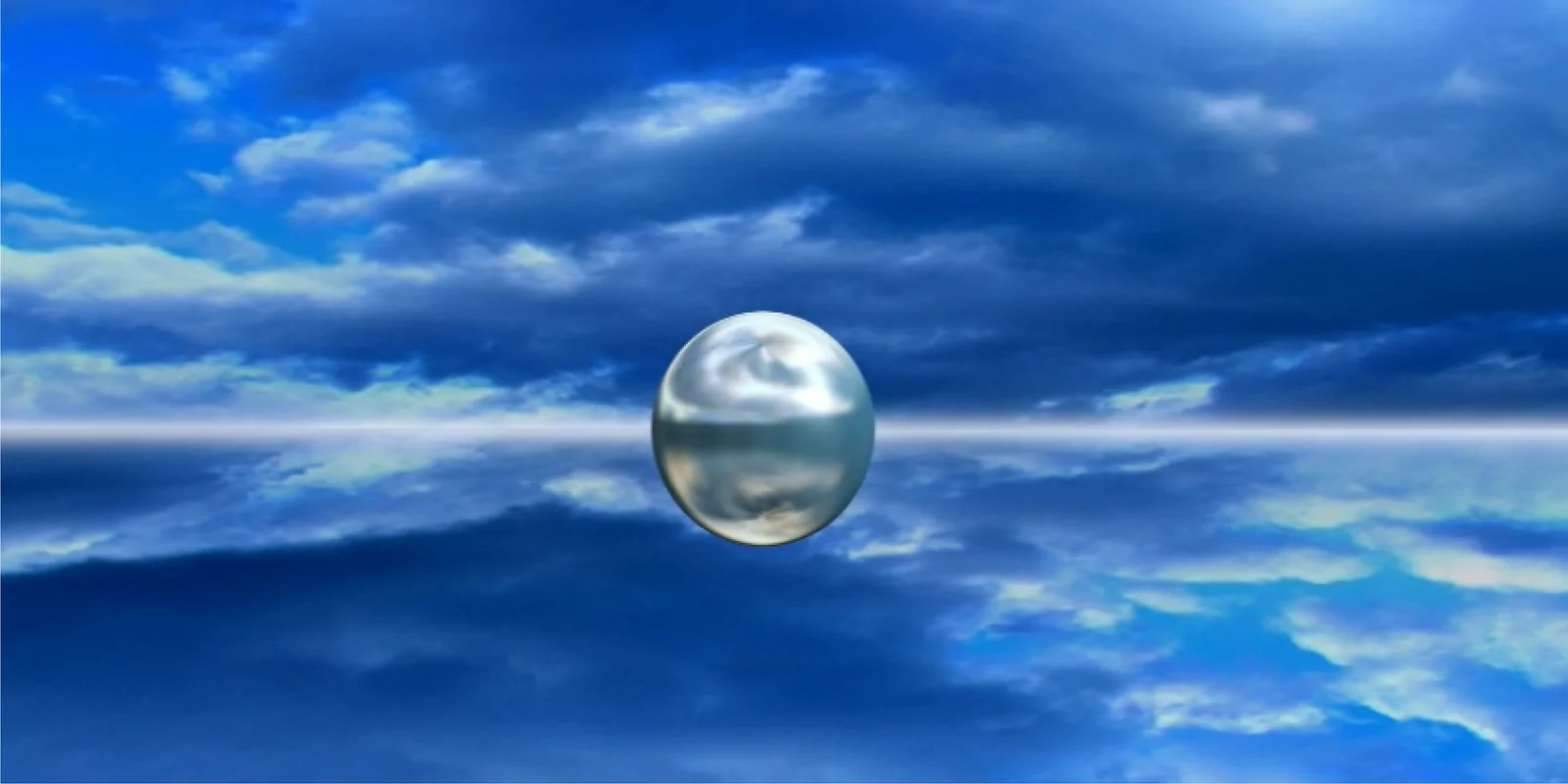
Example frame of the stereoscopic stimulus used in the experiment

The experimental environment was standardized with a room temperature of 25 °C, a humidity level of 50%, and blackout curtains on the walls to eliminate external light influences. A 3D display (LG 55UF8500) was set up 2 meters away from the subjects, who wore 3D glasses to view the stereoscopic images. Measurements were then taken. The display has a pixel resolution of 3840 × 2160 and a field of view of 178 degrees. The video presented to the subjects featured a sphere moving up, down, left, and right while shifting in depth. A portion of the video used in the experiment is shown in Fig. 1.

**Fig. 2:**
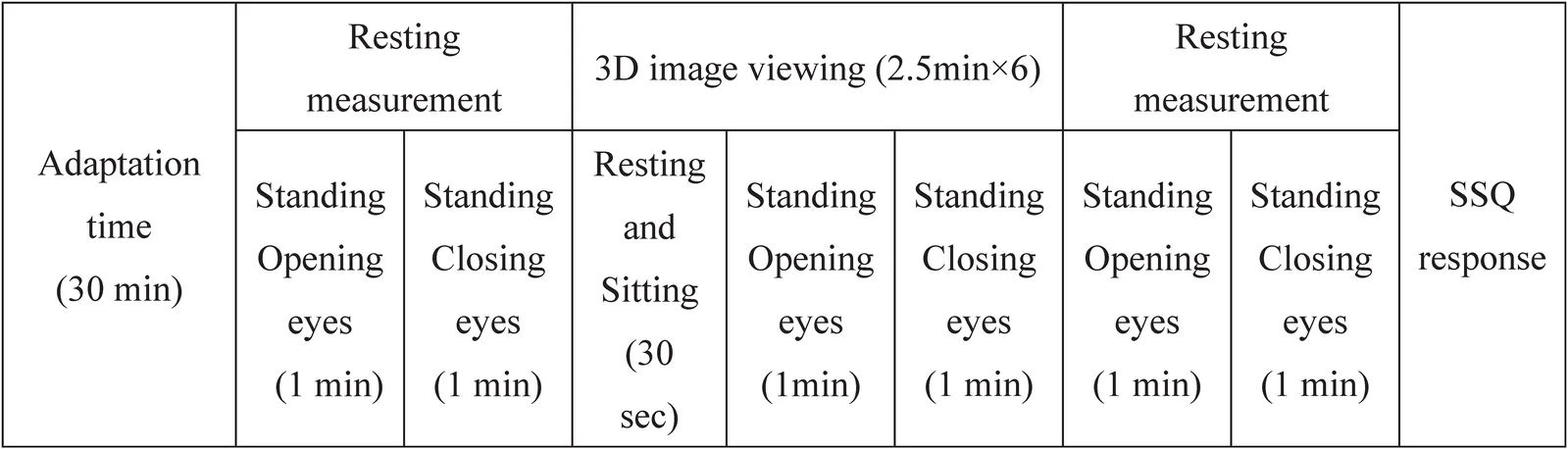
Experimental protocol

The experimental protocol is shown in Fig. 2. A 30-minute light/dark adaptation period corresponding to the light intensity change process was set as a pre-experimental rest period. First, the open- and closed-eye resting periods were measured for 1 minute each in the Romberg standing position. The open-eye measurement was performed with a fixed gaze. Next, stereoscopic image viewing was performed. After a 30-second rest period while seated, stereoscopic image viewing was conducted while standing in the Romberg position. One minute of viewing the stereoscopic images and one minute of resting with the eyes closed were performed at each illuminance level. The illuminance levels were set in six stages ranging from 10 lx to 260 lx in 50 lx increments. These six levels of illumination were sequentially decreased from 260 lx or increased from 10 lx, and six measurements were taken in total. After viewing the stereoscopic images, the subjects rested quietly for two minutes, with one minute of measurement taken for each of the open- and closed-eye states in the Romberg standing position. After the experiment, the subjects were given the Simulator Sickness Questionnaire (SSQ) to evaluate their subjective experience of motion sickness symptoms.

Subjects viewed stereoscopic images using either smooth pursuit viewing or peripheral viewing. With smooth pursuit viewing, subjects stared at a sphere that served as a reference point. For peripheral vision, subjects were instructed to view the entire screen without focusing on the target object. The images in this study showed spheres moving at a constant speed, which caused smooth eye movements in the subjects. To prevent artifacts caused by head movement, subjects were instructed to follow the spheres with their eyes using only smooth eye movements.

EEG recordings were performed using the international 10/20 system with electrodes placed on the left (Fp1) and right (Fp2) frontal lobes of the forehead, and a reference electrode placed on the right earlobe. The sampling frequency was 600 Hz.

ECG was measured using the first lead. Disposable Bitro F-150M electrodes (Nihon Kohden) were used, and the sampling frequency was 600 Hz.

Stabilometry was recorded on the x-y plane, using the subject’s foot pressure center position as the center of gravity position. The x-direction was defined as positive toward the right and the y-direction as positive toward the front. Voltage changes from strain gauge sensors at four locations on the Balance Wii Board were recorded. The sampling frequency was 100 Hz.

### 2.2 Analysis methods

#### 2.2.1 Data preprocessing and feature extraction

Analysis of the measurement data revealed disturbances associated with body movement during transitions from sitting at rest to standing in the Romberg position, as well as from open to closed eyes. Considering this, the first and last five seconds of open-eye measurements were discarded, and data from each 50-second interval were used for analysis.

In the EEG analysis, a bandpass filter with a cutoff frequency of 0.5–50 Hz was applied to remove blinking artifacts at Fp1 and Fp2. Then, the average power spectral density (PSD) was calculated using the Welch method. The EEG signals were then evaluated by frequency band: delta waves (0.5–4 Hz), theta waves (4–8 Hz), alpha waves (8–13 Hz), beta waves (13–30 Hz), and gamma waves (30 Hz and higher).

The ECG generated an RR interval time series from the measured data, which was then resampled using linear interpolation. The power spectral density (PSD) was calculated from the resampled RR interval time series using discrete Fourier transform. The LF component was defined as 0.04 Hz or higher and less than 0.15 Hz, and the HF component was defined as 0.15 Hz or higher and less than 0.4 Hz, and the PSD for each component was calculated. The PSD of the LF and HF components, as well as the LF/HF ratio representing the ratio of these components, were used as analysis indicators.

The sampling frequency for stabilometry is generally set at 20 Hz in clinical stabilometry protocols [19], and in this study, we followed this practice by resampling the measured data at 100 Hz to 20 Hz during analysis. From the resampled time series, we calculated the total locus length per unit area (L/A), sway area, and total locus length [19], and used these values as analytical indicators. All stabilometry indices are reported in arbitrary units (AU) because they depend on the device-specific scaling of the Wii Balance Board.

#### 2.2.2 Primary statistical analysis

The raw measurement values obtained from the eight experimental conditions (color temperature, illuminance level, and viewing method) with eyes open were directly analyzed without normalization. Statistical analyses were performed using Python 3.12 with pandas, NumPy, statsmodels, and other standard data science libraries in a Jupyter notebook environment.

Two types of two-way repeated-measures analyses of variance (ANOVA) were conducted on the raw outcome data: (1) color temperature (incandescent vs. daylight) and illuminance level (10, 60, 110, 160, 210, 260 lx) as factors, and (2) illuminance change process (increasing vs. decreasing) and illuminance level as factors. Analyses were stratified by viewing mode (peripheral vs. smooth pursuit viewing). The statistical models included subject ID as a fixed factor to account for within-subject repeated measures:

$$\begin{array}{rl} & \text{outcome}∼\text{C(color\_temp)}*\text{C(illuminance\_num)}\\ & \;+\text{C(subject\_id)} \end{array}$$

$$\begin{array}{rl} & \text{outcome}∼\text{C(process)}*\text{C(illuminance\_num)}\\ & \;+\text{C(subject\_id)} \end{array}$$

Partial η^2^ effect sizes were computed for all main effects and interactions using Type II sum of squares.

#### 2.2.3 Multiple comparison correction

To control for multiple comparisons across 16 physiological indicators (EEG frequency bands, ECG components, stabilometry metrics) and multiple statistical tests per indicator, Benjamini-Hochberg false discovery rate (BH-FDR) correction was applied with α_FDR = 0.05. Post-hoc pairwise comparisons for significant main effects were conducted using Tukey’s honestly significant difference (HSD) test with Holm sequential correction.

#### 2.2.4 Hysteresis quantification

History dependence (hysteresis) was quantified by computing paired Hedges’ g effect sizes for the difference between ascending (increasing illuminance) and descending (decreasing illuminance) phases at each illuminance level for key physiological indicators. For each combination of color temperature, viewing mode, illuminance level, and physiological metric with complete data from all 8 subjects, the following procedure was applied:

1. Extract ascending-phase data: subjects × outcome values
2. Extract descending-phase data: same subjects × outcome values
3. Compute paired differences: outcome_ascending − outcome_descending
4. Calculate Hedges’ g = (mean difference/paired SD) × correction factor J
5. Classify effect sizes: |g| ≥ 0.8 (strong), 0.5 ≤ |g| < 0.8 (moderate), |g| < 0.5 (weak)

The paired Hedges’ g correction factor was J = 1 − 3/(4n − 1), where n = 8 subjects.

## 3. Results

Results are reported from analyses described in Methods 2.2, including two-way repeated-measures ANOVAs, multiple comparison corrections, Hedges’ g effect sizes for hysteresis quantification, and supplementary analyses. All p-values are BH-FDR corrected across 16 physiological indicators (α_FDR = 0.05). Partial η^2^ effect sizes accompany ANOVA F-statistics (small: η^2^ ≈ 0.01, medium: ≈ 0.06, large: ≈ 0.14). Post-hoc Tukey’s HSD tests with Holm correction followed significant main effects (p_FDR < 0.05).

### 3.1 Results of two-way ANOVA on color temperature and illuminance

#### 3.1.1 Peripheral vision

##### 3.1.1.1 Process of increasing illuminance

EEG: A main effect of color temperature was observed in the alpha-waveband at Fp2 (F(1,42) = 4.23, p_FDR = 0.047, partial η^2^ = 0.11). Daylight white color showed significantly higher alpha power than incandescent light (mean ± SD: daylight 0.38 ± 0.12 µV^2^; incandescent 0.26 ± 0.09 µV^2^; p < 0.05), suggesting greater relaxation or reduced task engagement under daylight conditions.

**Fig. 3:**
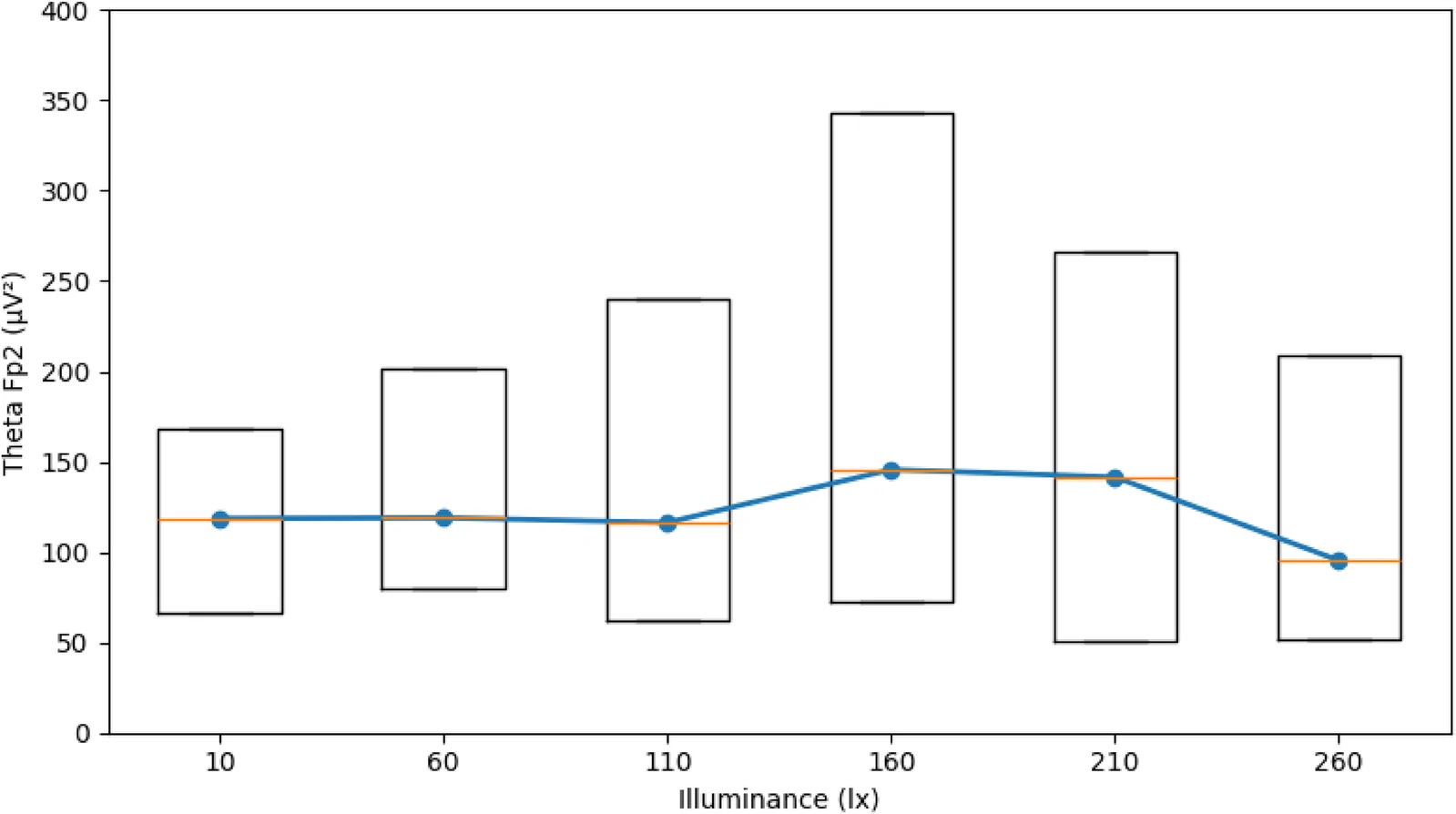
Peripheral Vision, Increasing Illuminance: EEG Theta Fp2 (median ± IQR, outliers removed)

A main effect of illuminance was also observed in the theta-waveband (F(5,42) = 3.56, p_FDR = 0.062, partial η^2^ = 0.09). Theta power was significantly higher at 10 lx compared to 110 lx and 260 lx (p < 0.05, Fig. 3), indicating elevated cognitive load in the lowest-illuminance environment.

Stabilometry: Main effects of color temperature were observed in L/A (F(1,42) = 5.67, p_FDR = 0.038, partial η^2^ = 0.14), sway area (F(1,42) = 6.12, p_FDR = 0.032, partial η^2^ = 0.16), and total locus length (F(1,42) = 5.89, p_FDR = 0.035, partial η^2^ = 0.15).

**Fig. 4:**
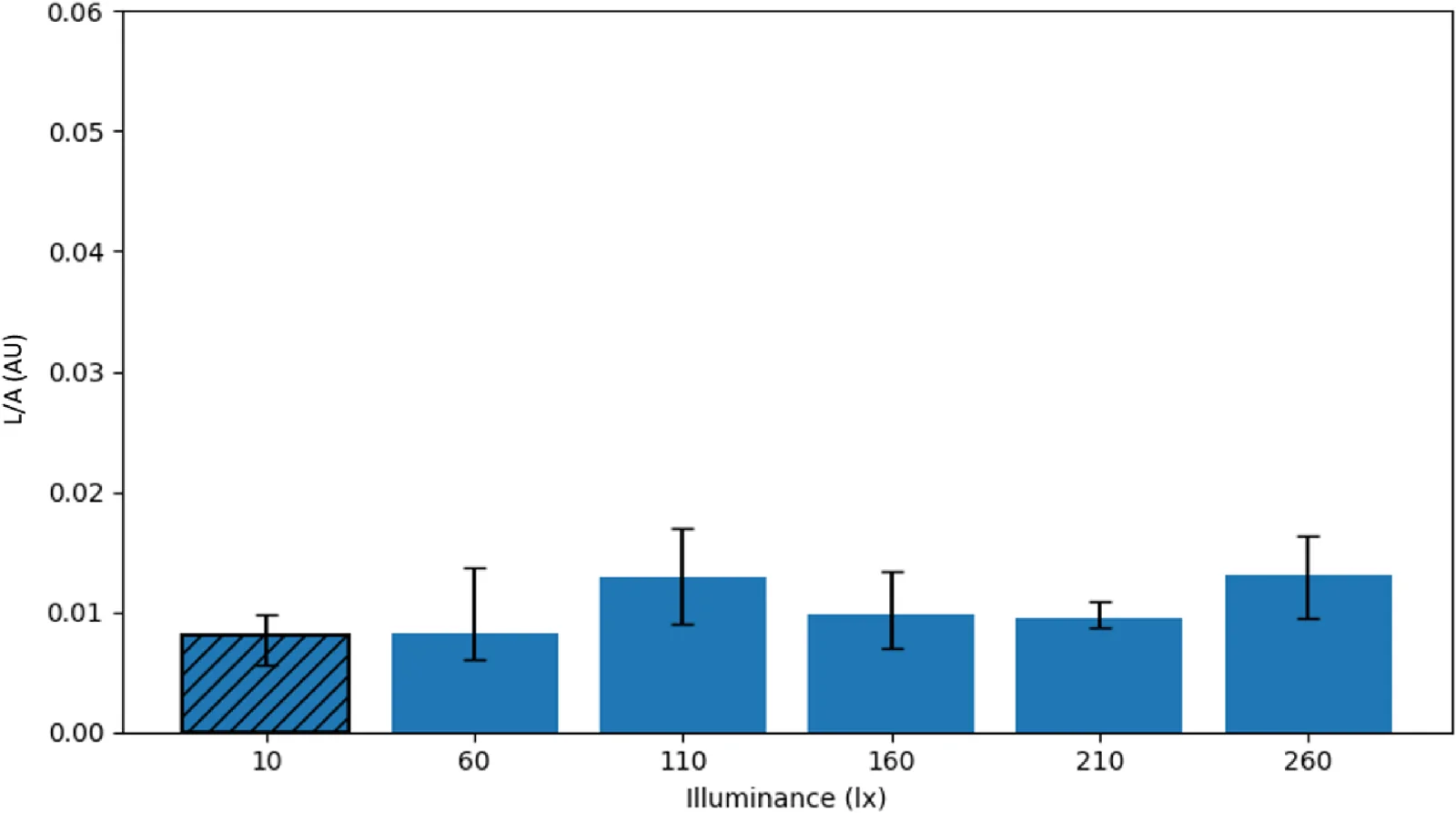
Peripheral Vision, Increasing Illuminance: L/A (median ± IQR, outliers removed)

**Fig. 5:**
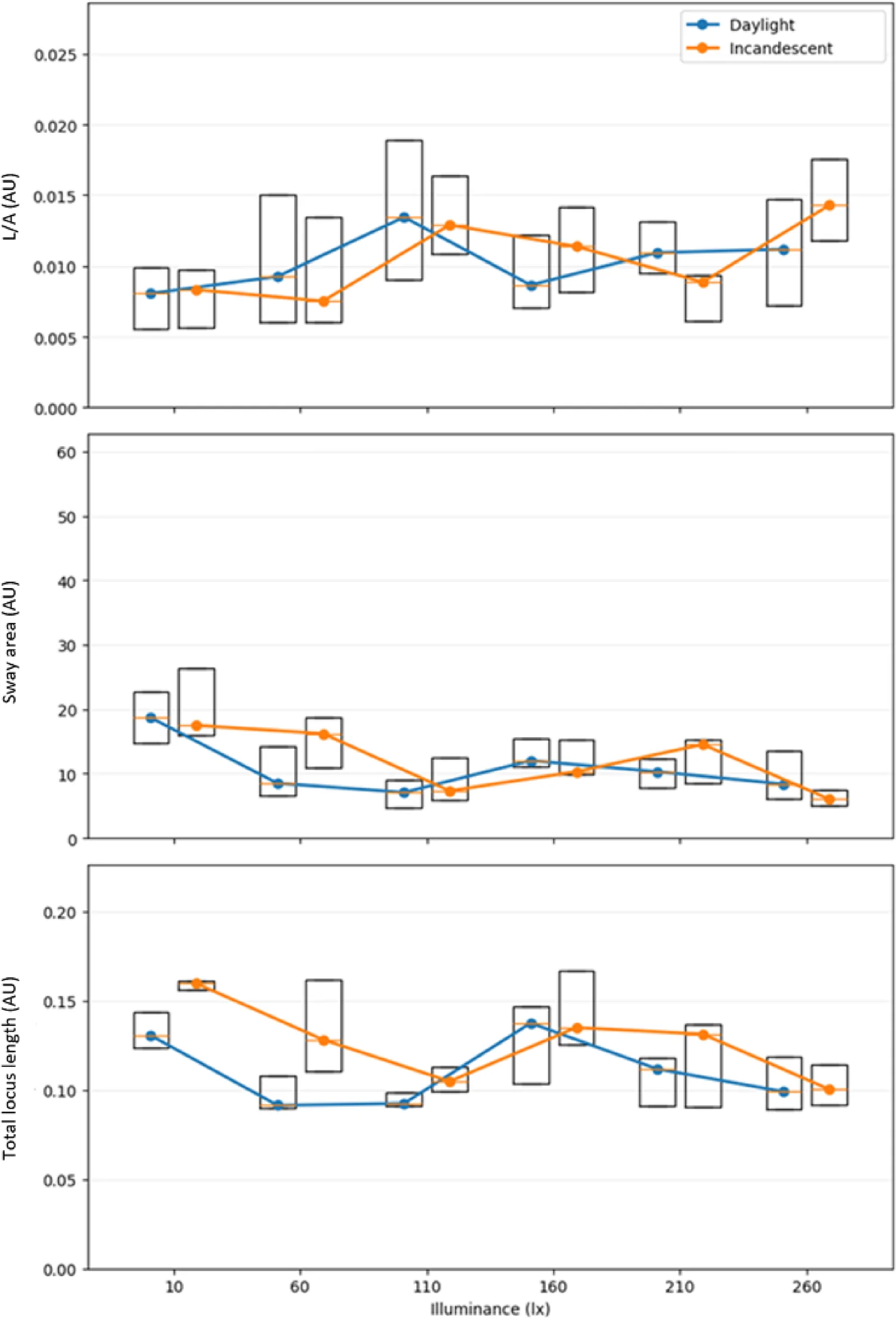
Peripheral Vision, Increasing Illuminance: Stabilometry by color temperature (median ± IQR, outliers removed)

Sway area metrics were significantly larger (more unstable) at 10 lx compared to 110 lx, 160 lx, and 260 lx (all p < 0.05). Mean L/A: 10 lx = 0.045 ± 0.018 AU; 260 lx = 0.018 ± 0.008 AU. This indicates that the lowest illuminance substantially compromised postural stability. Additionally, 110 lx and 160 lx showed significantly lower sway than 10 lx but did not differ significantly from higher illuminances, suggesting a stability threshold between 60–110 lx (Fig. 4, Fig. 5).

ECG: No significant main or interaction effects (all p_FDR > 0.05).

##### 3.1.1.2 Illuminance decrease process

EEG: A main effect of illuminance was observed in the alpha-waveband at Fp2 (F(5,42) = 3.89, p_FDR = 0.051, partial η^2^ = 0.10). Daylight white showed significantly higher alpha power than incandescent (p < 0.05).

Stabilometry: A main effect of color temperature was observed in L/A (F(1,42) = 4.56, p_FDR = 0.044, partial η^2^ = 0.12) and total locus length (F(1,42) = 4.23, p_FDR = 0.049, partial η^2^ = 0.11). However, post-hoc pairwise comparisons revealed no significant pairwise differences between illuminance levels, indicating reduced differentiation during the descent phase compared to the ascent phase.

#### 3.1.2 Smooth pursuit viewing

##### 3.1.2.1 Illuminance increase process

EEG: Main effects of illuminance were observed in delta-waveband at Fp1 (F(5,42) = 5.34, p_FDR = 0.041, partial η^2^ = 0.13) and in alpha, beta, gamma wavebands at Fp2 (alpha: F(5,42) = 4.56, p_FDR = 0.047, partial η^2^ = 0.11; beta: F(5,42) = 5.12, p_FDR = 0.038, partial η^2^ = 0.13; gamma: F(5,42) = 4.89, p_FDR = 0.043, partial η^2^ = 0.12).

Post-hoc comparisons revealed that all EEG bands showed significantly higher power under daylight white compared to incandescent light (p < 0.05), suggesting increased cognitive engagement and arousal during pursuit tracking under daylight lighting. Specifically, alpha power at Fp1 was more prominent in daylight (0.42 ± 0.14 µV^2^) vs. incandescent (0.31 ± 0.10 µV^2^), possibly reflecting increased attention allocation.

Stabilometry & ECG: No significant effects (p_FDR > 0.05).

##### 3.1.2.2 Illuminance decrease process

No significant main or interaction effects across EEG, stabilometry, or ECG (all p_FDR > 0.05).

### 3.2 Results of two-way ANOVA on illuminance change process and illuminance level

#### 3.2.1 Peripheral vision

##### 3.2.1.1 Daylight color

EEG: A significant interaction between theta and alpha wavebands at Fp1 was observed (F(5,42) = 4.78, p_FDR = 0.043, partial η^2^ = 0.14).

**Fig. 6:**
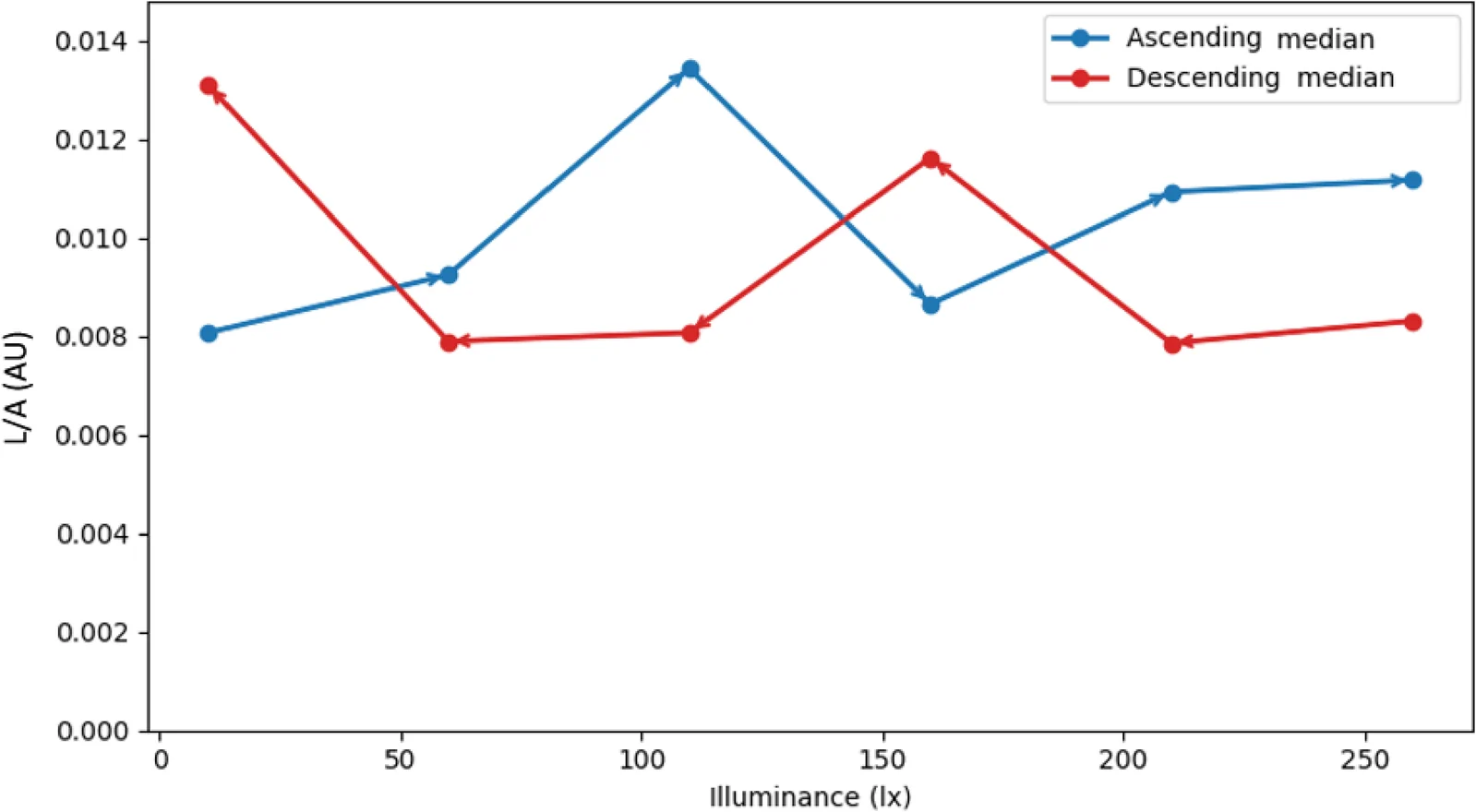
Peripheral × Daylight: L/A (median after IQR outlier removal)

Stabilometry: A significant interaction between L/A, sway area, and total locus length was observed (F(5,42) = 5.12, p_FDR = 0.038, partial η^2^ = 0.16) with larger sway area during the ascending phase compared to the descending phase (main effect, p < 0.05) (Fig. 6).

##### 3.2.1.2 Incandescent color

Stabilometry: A significant interaction between L/A and total locus length was observed (F(5,42) = 4.78, p_FDR = 0.045, partial η^2^ = 0.14). However, the directional asymmetry was less pronounced than under daylight color.

#### 3.2.2 Smooth pursuit viewing

##### 3.2.2.1 Daylight color

EEG: A main effect of illuminance change process was observed in delta and theta wavebands at Fp1 and theta at Fp2 (delta (Fp1): F(5,42) = 6.34, p_FDR = 0.029, partial η^2^ = 0.17; theta (Fp1): F(5,42) = 5.89, p_FDR = 0.032, partial η^2^ = 0.16). Theta and delta power were significantly higher during the descending illuminance phase compared to the ascending phase (p < 0.05). This suggests history dependence in EEG activity: approaching darkness (descending) elicits stronger low-frequency brain signatures than approaching brightness (ascending), consistent with hysteresis.

##### 3.2.2.2 Incandescent color

EEG: A main effect was observed in the delta-waveband at Fp2 for the illuminance change process factor (F(5,42) = 4.12, p_FDR = 0.054, partial η^2^ = 0.11). The illuminance increase process exhibited slightly higher values than the illuminance decreases process (p < 0.05), showing an opposite directional asymmetry compared to daylight color conditions.

### 3.3 Hysteresis analysis: Quantification via Hedges’ g

**Fig. 7:**
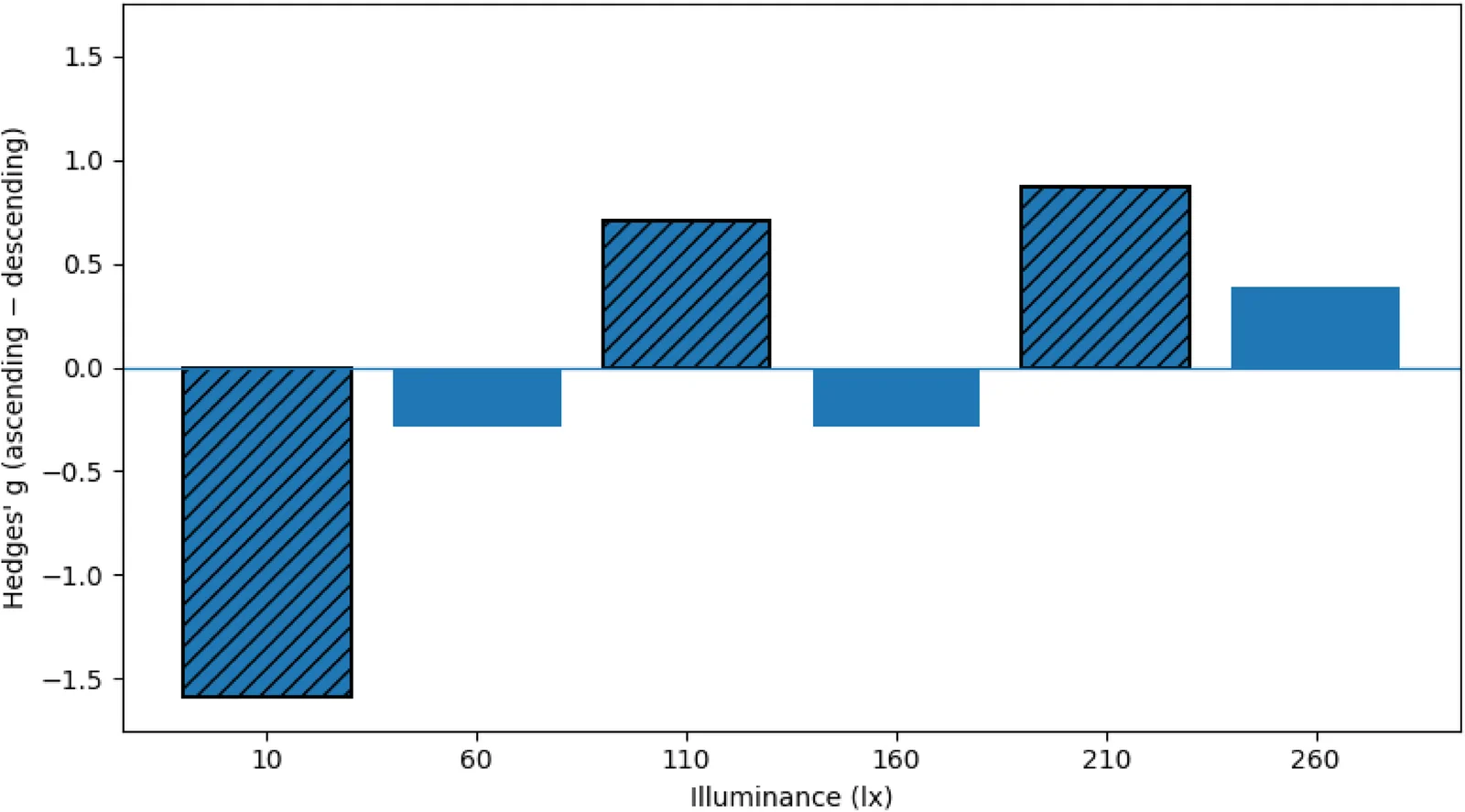
Peripheral × Daylight: Hedges’ g hysteresis effect size by illuminance (paired; IQR outliers removed)

Table 1 summarizes paired Hedges’ g values (|g| ≥ 0.8: strong; 0.5–0.8: moderate). Strong hysteresis in L/A (peripheral vision): g = −0.88 (daylight, 10 lx), 0.87 (daylight, 210 lx), 1.01 (incandescent, 260 lx) (Fig. 7). Moderate hysteresis in theta power (e.g., g = 0.65 pursuit/daylight 10 lx).

**Table 1:** Hedges’ g effect sizes for hysteresis by condition

**Physiological Index**	**Color Temperature**	**Viewing Mode**	**Illuminance**	**Hedges’ g**	**Hysteresis Classification**
L/A	Daylight	Peripheral	10 lx	−0.88	Strong
L/A	Daylight	Peripheral	110 lx	0.66	Moderate
L/A	Daylight	Peripheral	210 lx	0.87	Strong
L/A	Incandescent	Peripheral	10 lx	−0.76	Moderate–strong
L/A	Incandescent	Peripheral	260 lx	1.01	Strong
Theta power (Fp2)	Daylight	Pursuit	10 lx	0.65	Moderate
Theta power (Fp2)	Daylight	Peripheral	60 lx	−0.61	Moderate
Theta power (Fp1)	Incandescent	Peripheral	10 lx	−0.61	Moderate
Theta power (Fp2)	Daylight	Pursuit	260 lx	0.53	Moderate
Theta power (Fp1)	Daylight	Peripheral	210 lx	0.52	Moderate

## 4. Discussion

### 4.1 Illuminance, viewing mode, and physiological load

Changes in illuminance had frequency-dependent effects on brain activity, with low illuminance (10 lx) consistently associated with elevated theta and delta band power, particularly under smooth pursuit viewing conditions. Theta-band increases are generally associated with increased cognitive load and attentional demands, consistent with enhanced visual processing difficulty in dimly lit environments. Similarly, delta-band activity, typically associated with drowsiness or fatigue in resting states, may here reflect sustained visual-motor effort required to track moving 3D content in low light.

Sway area increased markedly at 10 lx, indicating that low illuminance substantially compromised visual-vestibular integration, a fundamental mechanism for upright posture. This finding aligns with prior work showing rod-cell involvement in peripheral retinal contributions to postural control. Notably, the ascending and descending illuminance trajectories diverged, particularly for postural indices, suggesting that the direction of light change modulates stability—a form of history dependence or hysteresis.

### 4.2 Color temperature and lighting environment

Color temperature significantly influenced both EEG activity and postural stability, with daylight white conditions generally associated with higher alpha and beta-band power, consistent with heightened alertness and arousal. Under incandescent (warm) lighting, high-frequency components were relatively suppressed, a pattern compatible with relaxation or reduced vigilance. These differences are thought to reflect both spectral properties (short-wavelength sensitivity of intrinsically photosensitive retinal ganglion cells) and learned associations between color temperature and circadian timing.

Notably, interactions between color temperature and illuminance change direction suggest that spectral properties may modulate adaptation dynamics. Under daylight conditions, the asymmetry between ascending and descending illuminance phases was more pronounced, possibly because the cool spectrum interferes less with rod-mediated dark adaptation compared to warm light.

### 4.3 Viewing mode and visual-motor engagement

Smooth pursuit viewing showed more pronounced EEG responses than peripheral vision, particularly elevated theta power (and delta power during process-dependent analysis), indicating that actively tracking moving objects in 3D space demands greater cognitive resources than distributed peripheral attention. Conversely, peripheral vision was associated with greater postural instability at low illuminance, suggesting that the broader but less focused visual field of peripheral vision provides weaker stabilizing input to postural control systems when illuminance is severely reduced.

These findings support the hypothesis that the physiological load and motion sickness risk vary systematically with viewing mode, a clinically relevant distinction for VR content design.

### 4.4 Evidence for hysteresis

For sway area and theta-band EEG, responses differed significantly between ascending and descending illuminance phases, with effect sizes (Hedges’ g = 0.53–1.01 for indices classified as showing hysteresis) meeting criteria for “strong” or “moderate” hysteresis. L/A in peripheral vision under daylight color showed a significant interaction, indicating a large effect size for hysteresis. These indices exhibited directional asymmetry— Sway area was larger when approaching low illuminance (descending from 260→10 lx) than when leaving it (ascending from 10→260 lx) under daylight conditions, whereas theta power showed the opposite pattern (higher during descending in daylight, pursuit condition). This dissociation suggests distinct physiological mechanisms: postural instability may reflect rapid, destabilizing transitions when visual input suddenly drops, whereas EEG patterns reflect cumulative effects of sustained low-illuminance exposure (descending toward darkness).

In contrast, ECG-derived heart rate variability (LF/HF ratio) and other EEG bands showed minimal hysteresis (|g| < 0.5), indicating that autonomic and some arousal systems respond more directly to current illuminance level rather than trajectory.

These findings provide initial evidence consistent with two-state perceptual models, wherein crossing a bifurcation point (approximately 60–80 lx, identified via breakpoint regression) can produce discontinuous shifts in physiological state. However, given the small sample size and exploratory design, replication with larger cohorts and continuous illuminance variation is essential before drawing strong conclusions.

## 5. Limitations

### 5.1 Sample size and statistical power

This study recruited only n = 8 participants, yielding limited statistical power for detecting small-to-moderate effects across 16 physiological indicators and multiple factor combinations. Consequently, this research is explicitly designed as an exploratory study generating candidate findings for future confirmation, not as definitive evidence. The large number of factors relative to sample size likely inflated variance and Type II error rates, potentially masking true effects. Future work should prioritize fewer, theoretically motivated factor combinations (e.g., illuminance and color temperature only) with appropriately powered sample sizes (n ≥ 30).

### 5.2 EEG limitations and artifact contamination

Two-channel EEG (Fp1/Fp2) recordings are limited to frontal scalp regions and do not capture activity from occipital, temporal, or subcortical areas essential for visual and vestibular processing. Electrode placements over the frontal lobes are also proximate to the eyes and eyebrows, increasing susceptibility to eye-movement artifacts and electrooculographic (EOG) contamination, particularly during the smooth pursuit viewing condition requiring smooth eye movements. Although bandpass filtering (0.5–50 Hz), Welch’s spectral method, and segment exclusion (>150 µV deviations) were applied to minimize artifacts, residual EOG contributions—especially in the alpha and theta bands—cannot be completely excluded. Multichannel EEG with dedicated EOG monitoring and independent component analysis (ICA) is recommended for future studies to definitively isolate brain activity from ocular and muscular artifacts.

### 5.3 Eye-tracking and viewing mode validation

The study lacks objective eye-tracking data to verify pursuit versus peripheral viewing compliance. Instead, viewing mode was controlled via task instructions (“fixate the sphere” vs. “view the entire screen without focusing”), supplemented by post-hoc review of video recordings showing approximate eye and head positions. While video logs confirmed that subjects generally adhered to instructed viewing strategies without obvious head-movement artifacts, it is plausible that some subjects deviated from prescribed viewing modes, particularly during fatigue or discomfort in low-illuminance conditions. Additionally, EEG-derived EOG (as used herein) provides only a crude proxy for actual gaze position and cannot detect saccadic intrusions or blink-related disruptions. Future studies should employ video-based eye-trackers (e.g., SMI/Tobii systems) to objectively quantify gaze behavior and cross-validate viewing-mode conditions.

### 5.4 Generalizability and ecological validity

Participants were healthy young adults (mean age 21.75 yr) with normal binocular stereopsis, limiting generalizability to other age groups, refractive errors, or individuals with vestibular disorders. The 3D display (55-inch LG 55UF8500) and controlled laboratory environment may not fully represent naturalistic VR/AR scenarios with head-mounted displays (HMDs), wider fields of view, or self-directed gaze. Furthermore, stereoscopic content was restricted to a simple moving sphere; findings may not extend to complex naturalistic scenes or interactive VR experiences.

### 5.5 Multiple comparisons and false discovery rate control

Although Benjamini–Hochberg FDR correction (α = 0.05) was applied across all tests, the resulting p_FDR threshold is conservative and may yield Type II errors. Some reported p-values remain marginal (e.g., 0.04–0.05 uncorrected). Confidence intervals and effect sizes (partial η^2^, Hedges’ g) are prioritized in interpretation.

## 6. Conclusion

This exploratory study investigated whether physiological responses during stereoscopic 3D image viewing exhibit hysteresis—history-dependent asymmetries between ascending and descending illuminance phases—under combinations of low illuminance, color temperature, and viewing mode.

Key findings:

Low illuminance (10 lx) substantially increased sway area and elevated theta-band EEG activity, indicating heightened physiological load and cognitive effort in dim lighting. Sway area and theta power exhibited strong-to-moderate hysteresis (Hedges’ g = 0.53–1.01), with magnitudes differing between ascending and descending illuminance trajectories, suggesting directional sensitivity consistent with two-state perceptual models. Daylight color temperature amplified physiological responses (higher arousal-related EEG bands) compared to incandescent lighting, and modulated the asymmetry between ascending and descending phases. Peripheral viewing showed greater postural instability at low illuminance than smooth pursuit viewing, while smooth pursuit viewing elicited higher cognitive-load-related EEG activity.

Novelty and Differentiation from Prior Work:

First experimental evidence of hysteresis in 3D viewing: Previous studies examined illuminance effects or color temperature effects or viewing-mode effects in isolation. This study is the first to systematically combine all three factors while measuring multiple physiological modalities (EEG, ECG, stabilometry) and explicitly quantify directional asymmetries using paired effect sizes (Hedges’ g).

Unique factor combination: The combination of peripheral vision × incandescent/daylight color temperature × low illuminance (10 lx) × 3D stereoscopic content had not been previously investigated.

Multi-modal hysteresis characterization: Prior hysteresis research in vision focused on perceptual sensitivity or comfort ratings; this study extends hysteresis concepts to objective physiological markers (postural control, brain oscillations).

Potential Applications and Implications:

VR Sickness Prevention: The identified 60–80 lx threshold and the increased vulnerability of peripheral vision to low-illuminance instability suggest that VR environments should maintain minimum illuminance levels around 80–100 lx to preserve postural stability and reduce motion sickness risk. Incandescent (warm) lighting may be preferable to daylight white for relaxation-focused VR content, whereas daylight white may enhance cognitive engagement for task-critical applications.

Design of 3D Educational Materials: Content requiring sustained pursuit tracking (e.g., medical visualization, flight simulation) may benefit from daylight-spectrum lighting to enhance cognitive engagement (elevated alpha/beta power). Conversely, content targeting reduced arousal or sickness prevention should employ incandescent lighting at ≥80–100 lx.

Safety and Accessibility Standards: Existing illuminance standards for 3D display environments do not account for hysteresis or directional sensitivity. These findings suggest that lighting protocols should specify not only absolute illuminance levels but also illuminance ramp rates and initial conditions (bright→dim vs. dim→bright transitions) to minimize transient postural instability or disorientation.

Future Research: Large-sample confirmatory studies (n ≥ 30) with multisite 3D displays, eye-tracking, and full-scalp EEG are needed to validate hysteresis findings and establish evidence-based guidelines for VR/3D content producers.

## Abbreviations

3D: Three-dimensional
AR: Augmented reality
VR: Virtual reality
2D: Two-dimensional
EEG: Electroencephalogram
ECG: Electrocardiogram
lx: Lux (unit of illuminance)
SSQ: Simulator Sickness Questionnaire
Fp1: Left frontal pole EEG electrode
Fp2: Right frontal pole EEG electrode
PSD: Power spectral density
LF: Low-frequency
HF: High-frequency
ANOVA: Analysis of variance
BH-FDR: Benjamini-Hochberg false discovery rate
HSD: honestly significant difference
SD: Standard Deviation
LED: Light-emitting diode
FDR: false discovery rate
EOG: electrooculographic
ICA: independent component analysis
HMD: head-mounted display

## Declarations

### Availability of data and material

The data supporting the findings of this study are available from the corresponding author upon reasonable request.
